# Supplementary material for: Evolutionary Patterns of Sex-Biased Genes in Three Species of Haplodiploid Insects
Source: Insects. 2020 May 26;11(6):326. doi: 10.3390/insects11060326 (PMC7349267; doi:10.3390/insects11060326)
Supplement: Supplementary file 1 [file insects-11-00326-s001.zip › Si-revision.docx]

Figure legends

Fig. S1. **Phylogeny trees of gene expression based on pairwise distance matrices** (1-ρ, ρ is Spearman’s correlation coefficient) built by NJ method across the three *Bemisia* species and the greenhouse whitefly (a), and across the three *Bemisia* species (b) (F = female; M = male). In the nodes of phylogentic trees, only the the bootstrap values less than 90 were labled.

Fig. S2. **Distributions of the gene number and the Ka/Ks ratio in gene expression variation in sexes.** Blue lines indicate median of the Ka/Ks ratio in each of the species-combinations. Significant differences of the Mann–Whitney test for each gene category compared with median levels: * P < 0.05, ** P < 0.01, *** P < 0.001, **** P < 0.0001.

Fig. S3. **Distributions of Dx, Vx and Rx in gene expression variation in sexes.** Blue lines indicate the median of the statistics presented in each of the three diagrams. Significant differences of Mann–Whitney test for each gene category compared with median levels: * P < 0.05, ** P < 0.01, *** P < 0.001, **** P < 0.0001.

Fig. S4. **The evolution of protein coding sequences and gene expressions in each of the detailed categories**. Number of genes (**a**), evolution of protein coding sequences (the Ka/Ks ratio) (**b**), gene variations across species (Dx) (**c**), gene variations within MEAM1 species (Vx) (**d**), evolution of gene expression (Rx) (**e**) in each of the gene categories. The dash line in each of the five diagrams indicates the median of all genes. Q = MED, B = MEAM1, Z = Asia II 3; Sex = sex-variable genes; Sp = speices-variable genes; Sex + Sp = sex- and species-variable genes; Int = interaction-variable genes; non-var=non-variable genes; F = female-biased; M = male-biased. Significant differences of the Mann–Whitney test for each gene category compared with median levels: ns P > 0.05, * P < 0.05, ** P < 0.01, *** P < 0.001, **** P < 0.0001.

Tables

**Table S1. Summary for transcriptome assembly of MEAM1, MED and Asia II 3 whiteflies.**

|  | **MEAM1** | **MED** | **Asia II 3** |
| --- | --- | --- | --- |
| Total clean reads | 181,966,344 | 102,079,564 | 189,043,732 |
| Read length (bp) | 100 | 100 | 100 |
| Total number of transcripts | 87,790 | 74,511 | 92,296 |
| Total numbers of unigenes | 63,020 | 46,805 | 59,120 |
| N50 of unigenes (bp)  Numbers of unigenes with NR annotation | 1,719  14,592 | 2,319  12,795 | 1,823  14,408 |

**Table S2 Statistics of gene expression data through mapping to the transcriptome of each species.**

|  |  | Clean reads | All reads mapping to genes | | | All tag-mapped genes | | | |
| --- | --- | --- | --- | --- | --- | --- | --- | --- | --- |
| Species | Samples | Total No. of reads | No. of reads | Percentage of reads mapping to genes | | No. of genes | | Percentage of tag-mapped genes |  |
| MEAM1 | B1f | 10,569,798 | 9,501,552 | 89.9% | 46,601 | | 73.9% | | |
|  | B1m | 11,193,563 | 9,846,113 | 88.0% | 47,551 | | 75.5% | | |
|  | B2f | 13,253,317 | 11,891,678 | 89.7% | 48,951 | | 77.7% | | |
|  | B2m | 11,437,476 | 10,146,654 | 88.7% | 49,468 | | 78.5% | | |
|  | B3f | 12,124,079 | 10,853,102 | 89.5% | 47,226 | | 74.9% | | |
|  | B3m | 11,247,703 | 9,934,548 | 88.3% | 48,565 | | 77.1% | | |
|  | B4f | 13,034,288 | 11,670,817 | 89.5% | 47,255 | | 75.0% | | |
|  | B4m | 9,716,051 | 8,557,241 | 88.1% | 48,092 | | 76.3% | | |
| MED | Q1f | 12,715,283 | 11,260,167 | 88.6% | 38,661 | | 82.6% | | |
|  | Q1m | 14,432,124 | 12,670,096 | 87.8% | 39,169 | | 83.7% | | |
|  | Q2f | 14,272,090 | 12,492,746 | 87.5% | 38,870 | | 83.0% | | |
|  | Q2m | 12,006,979 | 10,534,132 | 87.7% | 38,506 | | 82.3% | | |
| Asia II 3 | Z1f | 13,923,444 | 12,315,440 | 88.5% | 46,804 | | 79.2% | | |
|  | Z1m | 11,947,214 | 10,499,802 | 87.9% | 45,222 | | 76.5% | | |
|  | Z2f | 13,627,087 | 12,219,464 | 89.7% | 45,263 | | 76.6% | | |
|  | Z2m | 10,665,898 | 9,418,708 | 88.3% | 41,468 | | 70.1% | | |
| GW | T1f | 12,701,076 | 8,639,375 | 68.0% | 45,431 | | 83.0% | | |
|  | T1m | 11,022,874 | 7,819,621 | 70.9% | 43,464 | | 79.4% | | |
|  | T2f | 12,741,721 | 9,030,302 | 70.9% | 45,915 | | 83.9% | | |
|  | T2m | 13,387,713 | 9,049,036 | 67.6% | 45,656 | | 83.4% | | |

In the column of samples, ‘Q’, ‘B’, ‘Z’ and ‘T’ stand for MED, MEAM1, Asia II 3 whiteflies, and the greenhouse whitefly GW. ‘f’ and ‘m’ stand for female and male.

**Table S3** **Correlations of biological replication of each sample (R^2^).**

|  | female | male |
| --- | --- | --- |
| MED | 0.906 | 0.915 |
| Asia II 3 | 0.936 | 0.935 |
| MEAM1 population 1 | 0.829 | 0.885 |
| MEAM1 population 2 | 0.878 | 0.893 |

**Table S4 The number of sex-biased expression genes in the three *Bemisia* species.**

|  |  | **Female-biased** |  | **Non-sex-biased** | **Male-biased** |
| --- | --- | --- | --- | --- | --- |
| MEAM1 | No. of genes | 9,188 (15.7%) |  | 40,335 (68.8%) | 9,113 (15.5%) |
|  | No. genes with annotation | 2,769 (20.1%) |  | 8,778 (63.7%) | 2,233 (16.2%) |
| MED | No. of genes | 7,231 (16.5%) |  | 29,774 (67.7%) | 6,950 (15.8%) |
|  | No. of genes with annotation | 3,810 (30.7%) |  | 6,612 (53.3%) | 1,983 (16.0%) |
| Asia II 3 | No. of genes | 7,395 (14.3%) |  | 38,367 (74.4%) | 5,792 (11.2%) |
|  | No. of genes with annotation | 3,506 (27.0%) |  | 8,252 (63.5%) | 1,239 (9.5%) |

Sex-biased expression genes are based on threshold |Log_2_FC| > 1 and FDR < 0.05.

Proportion calculated according to the category in row.

**Table S5 Statistics of Ka/Ks between each pair of two species among orthologs.**

|  | No. genes of Ka/Ks > 1 | No. genes of Ka/Ks = 0 | No. gene of Ka/Ks < 0.1 | mean Ka/Ks | median Ka/Ks | | first quartile Ka/Ks | | third quartile Ka/Ks |
| --- | --- | --- | --- | --- | --- | --- | --- | --- | --- |
| MEAM1-MED | 98(1.8%) | 2,024(36.9%) | 2,953 (53.8%) | 0.170 | 0.0530 | | 0 | | 0.225 |
| MEAM1-Asia II 3 | 65(1.2%) | 1,231(22.4%) | 2,862 (52.2%) | 0.167 | 0.0895 | 0.0179 | | 0.234 | |
| MED - Asia II 3 | 63(1.1%) | 1,304(23.8%) | 2,914 (53.1%) | 0.162 | 0.0850 | 0.0136 | | 0.224 | |
| Average | 41(0.8%) | 904(18.3%) | 2,525 (51.1%) | 0.166 | 0.0963 | 0.0162 | | 0.227 | |

Proportion calculated according to each species.

Additional file

Additional file 1. GO and KEGG enrichment of sex-biased genes in MED.

Additional file 2. GO and KEGG enrichment of evolution of protein coding sequences, ‘high KaKs’ stands for upper quartile of Ka /Ks ratio ( > 0.227). ‘low Ka/Ks’ stands for the lower quartile of Ka /Ks ratio ( < 0.024).

Additional file 3. GO and KEGG enrichment of evolution of gene expression. ‘high Dx’ and ‘high Vx’ stand for >50% percentile in Dx and Vx, respectively. ‘low Dx’ and ‘low Dx’ stand for <50% percentile in Dx and Vx, respectively.

Additional file 4. GO and KEGG enrichment in each category separated by ANOVA.

Additional file 5. Average Ka/Ks, Rx, Dx and Sx in each KEGG pathway.
