# Supplementary figures and images for: Evolutionary Patterns of Sex-Biased Genes in Three Species of Haplodiploid Insects
Source: Insects. 2020 May 26;11(6):326. doi: 10.3390/insects11060326 (PMC7349267; doi:10.3390/insects11060326)

(a)

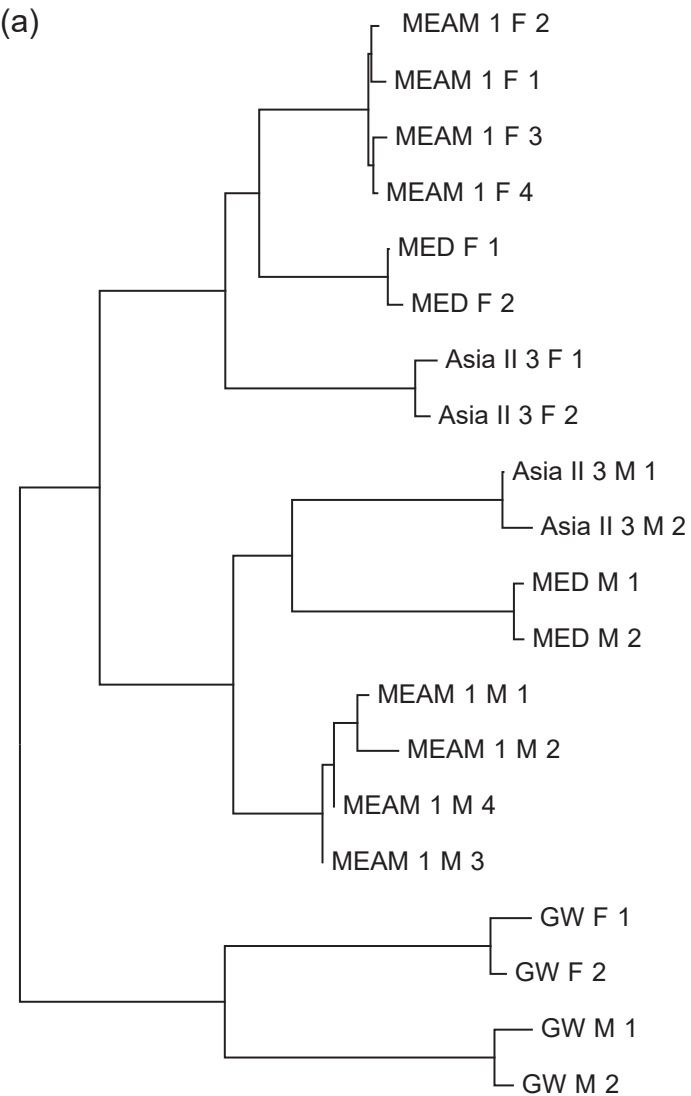

(b)

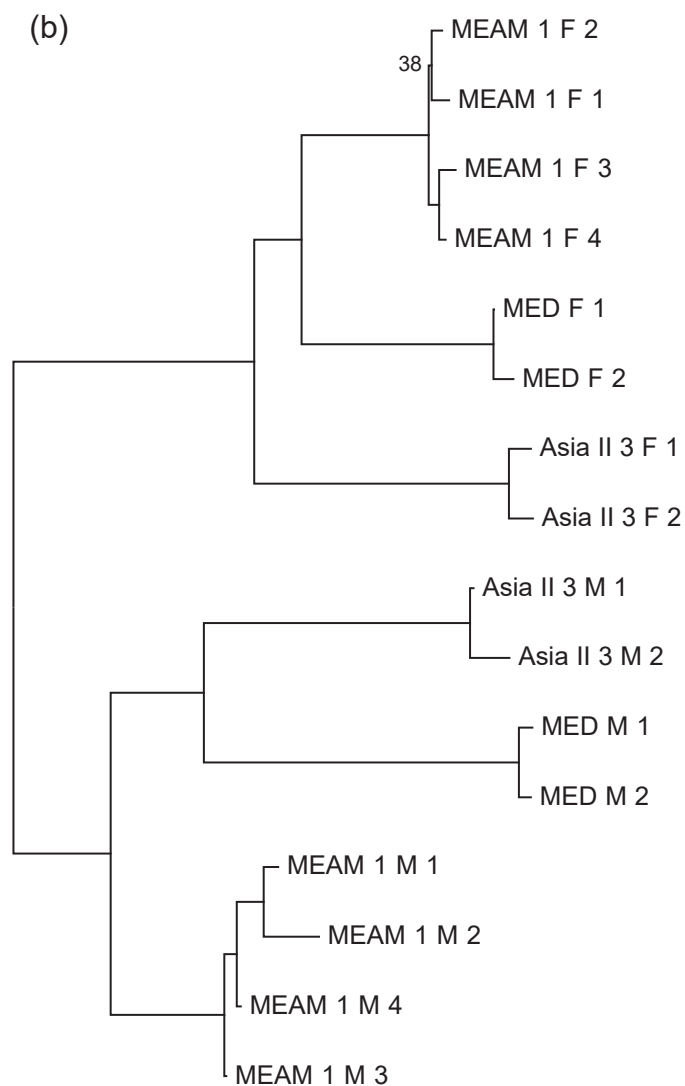

Supplement: Supplementary file 1 [file insects-11-00326-s001.zip › Fig. S1.pdf]

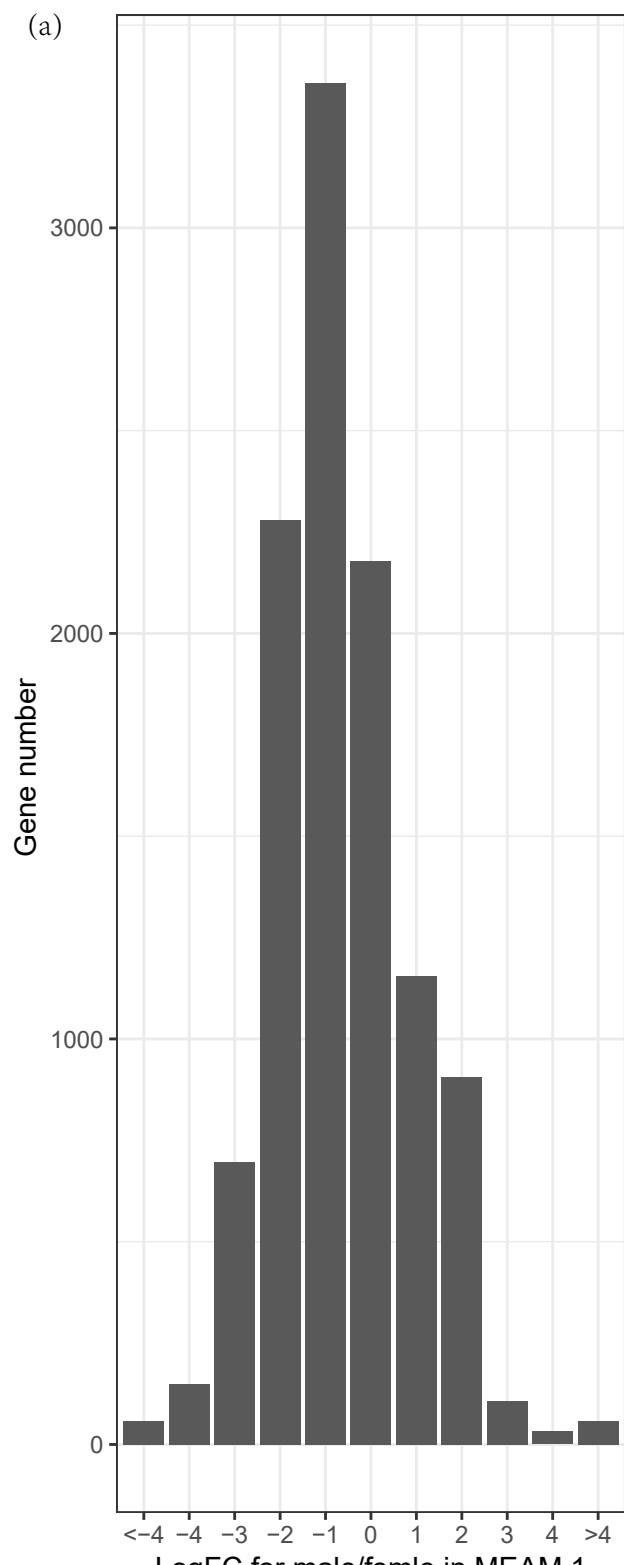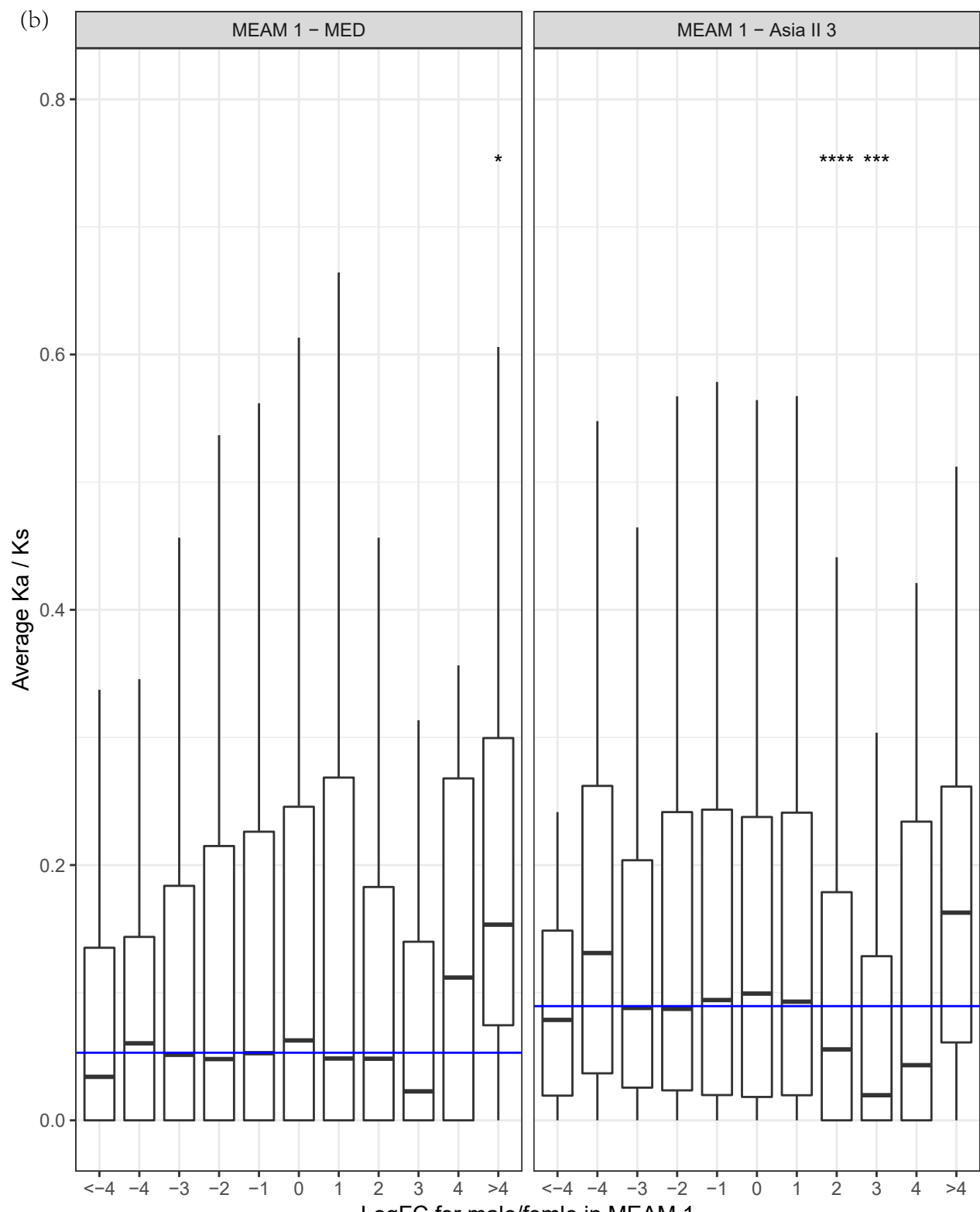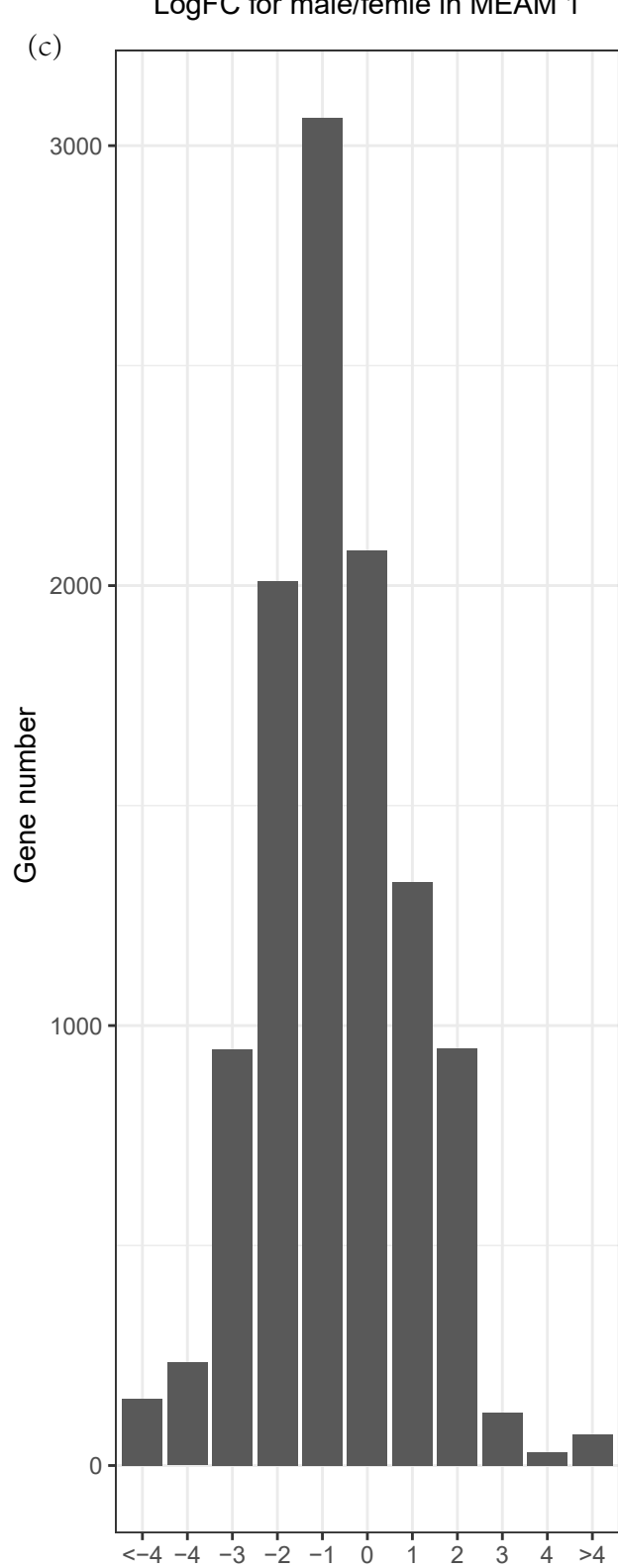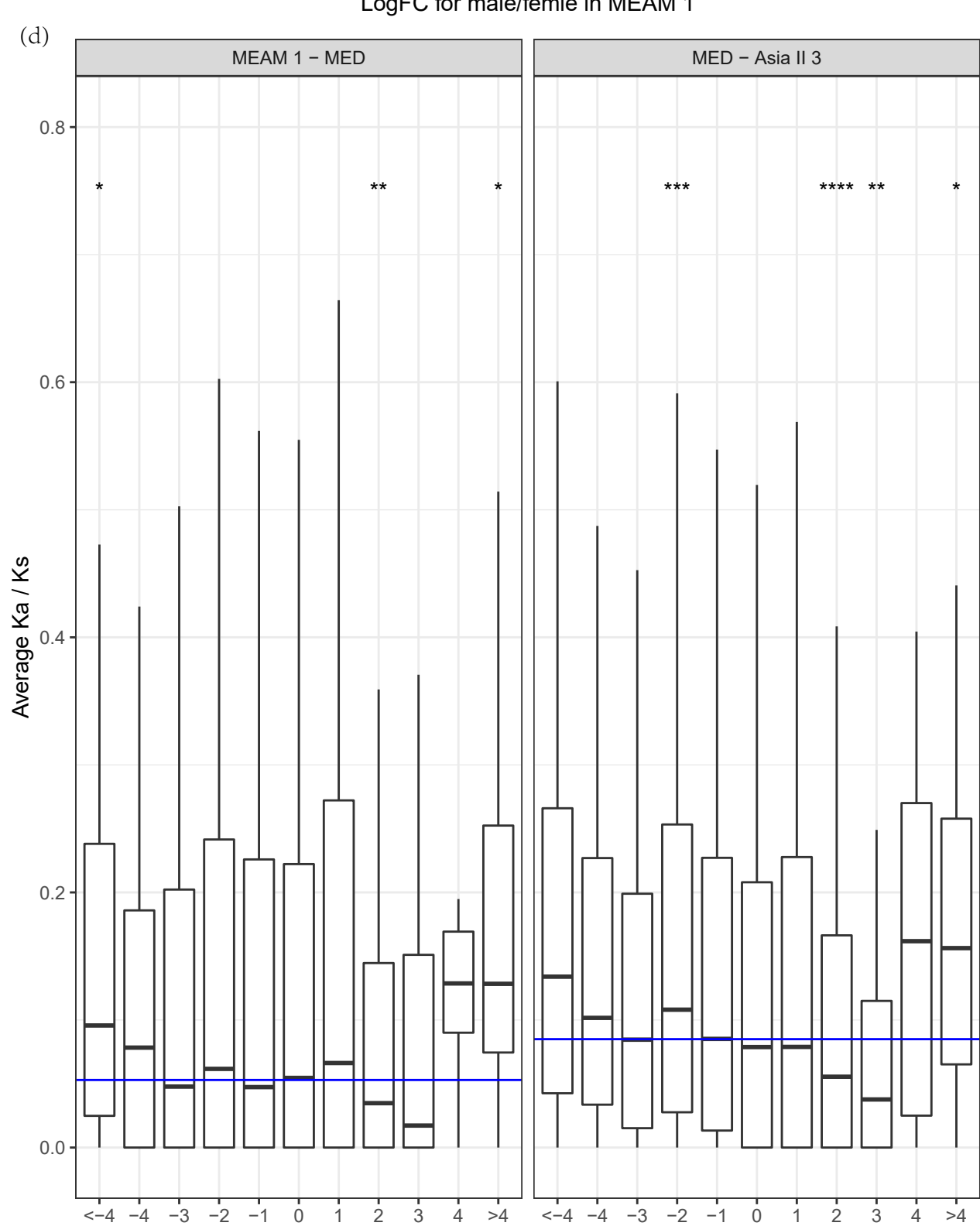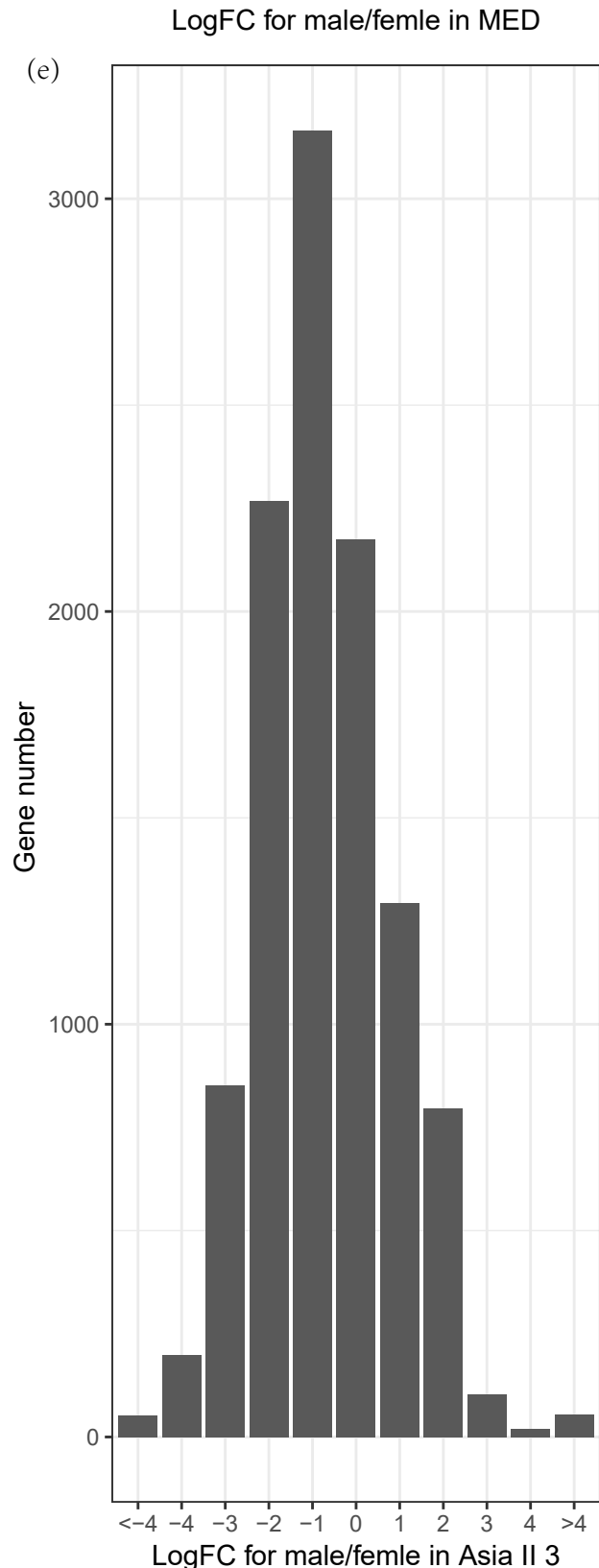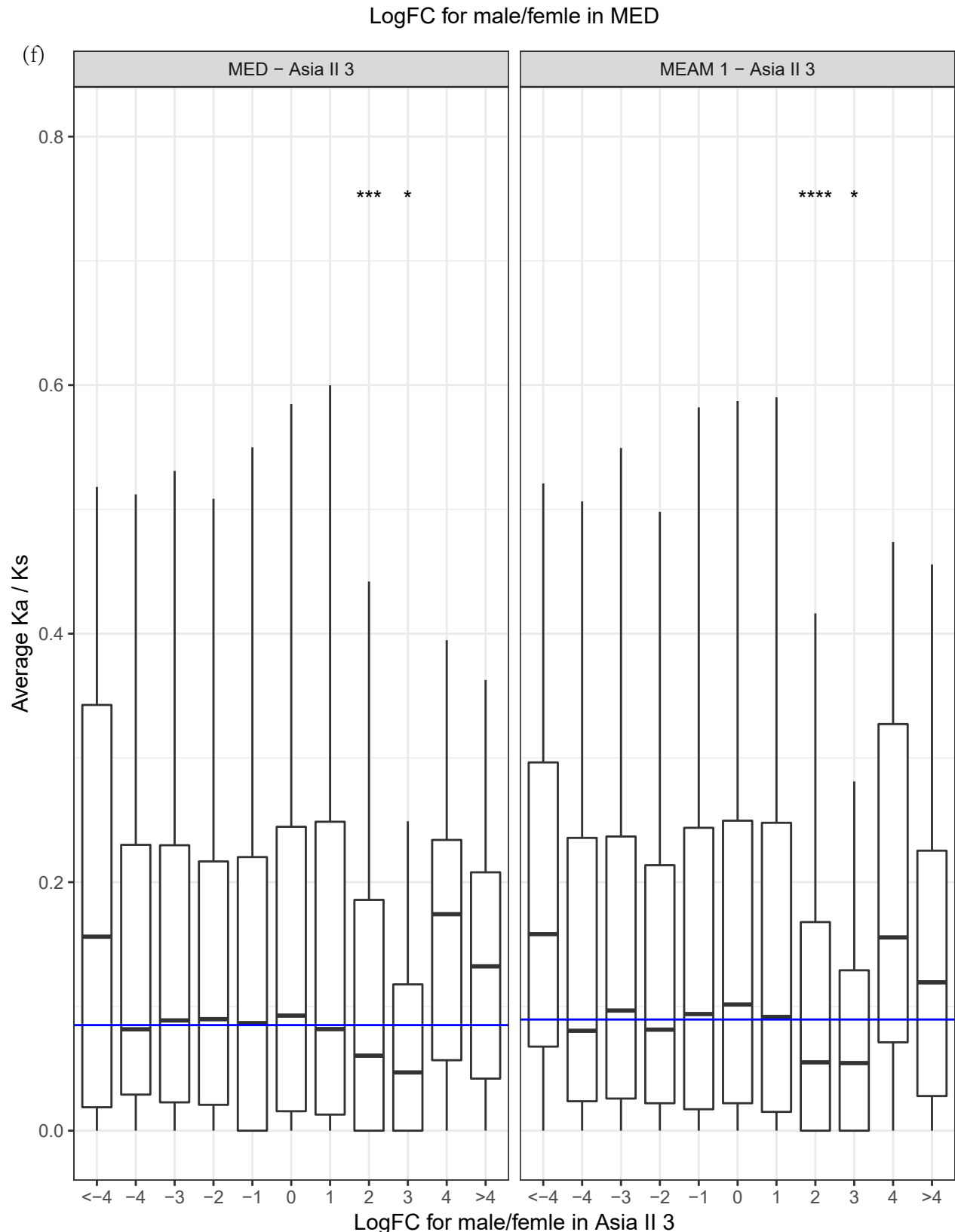

Supplement: Supplementary file 1 [file insects-11-00326-s001.zip › Fig. S2.pdf]

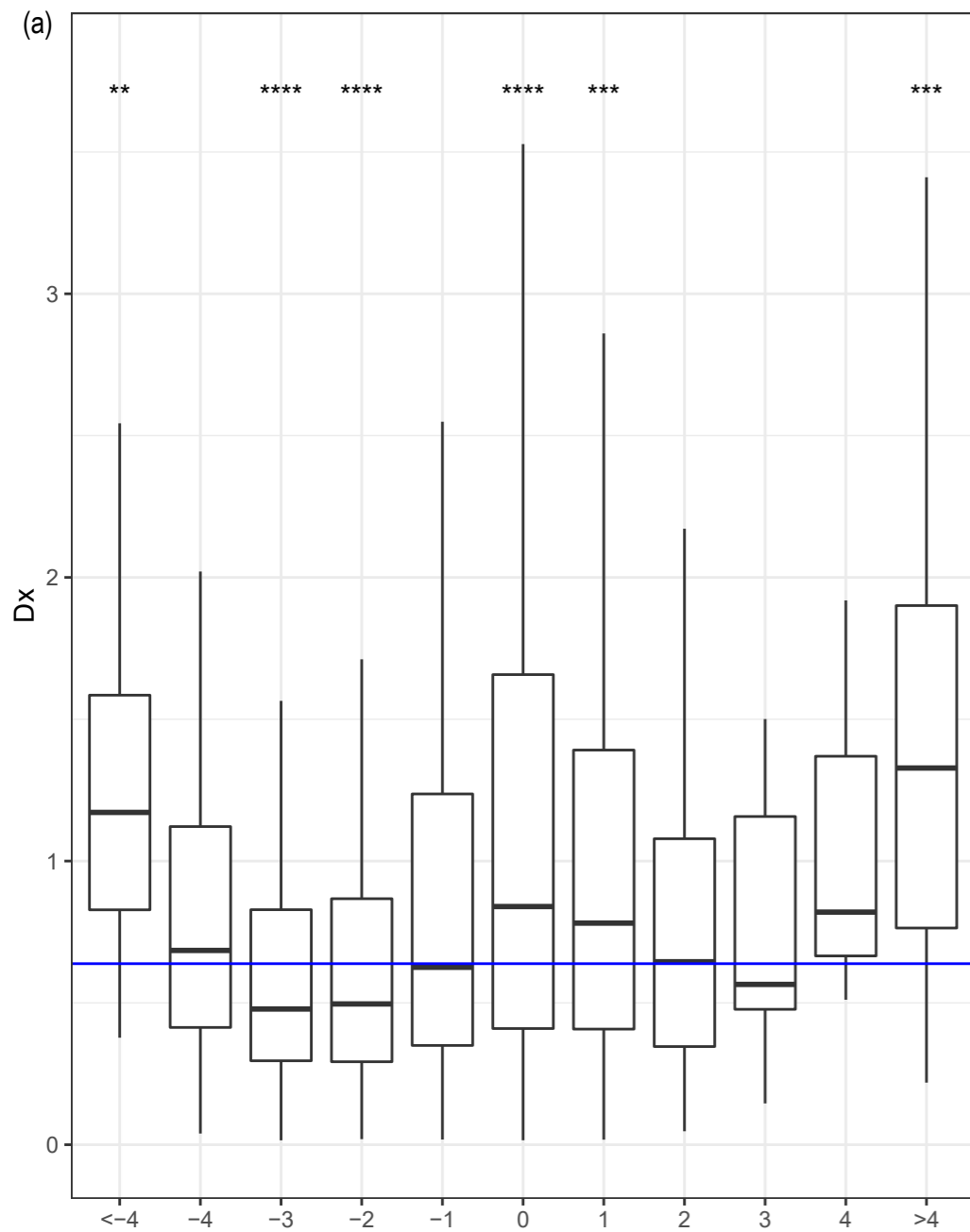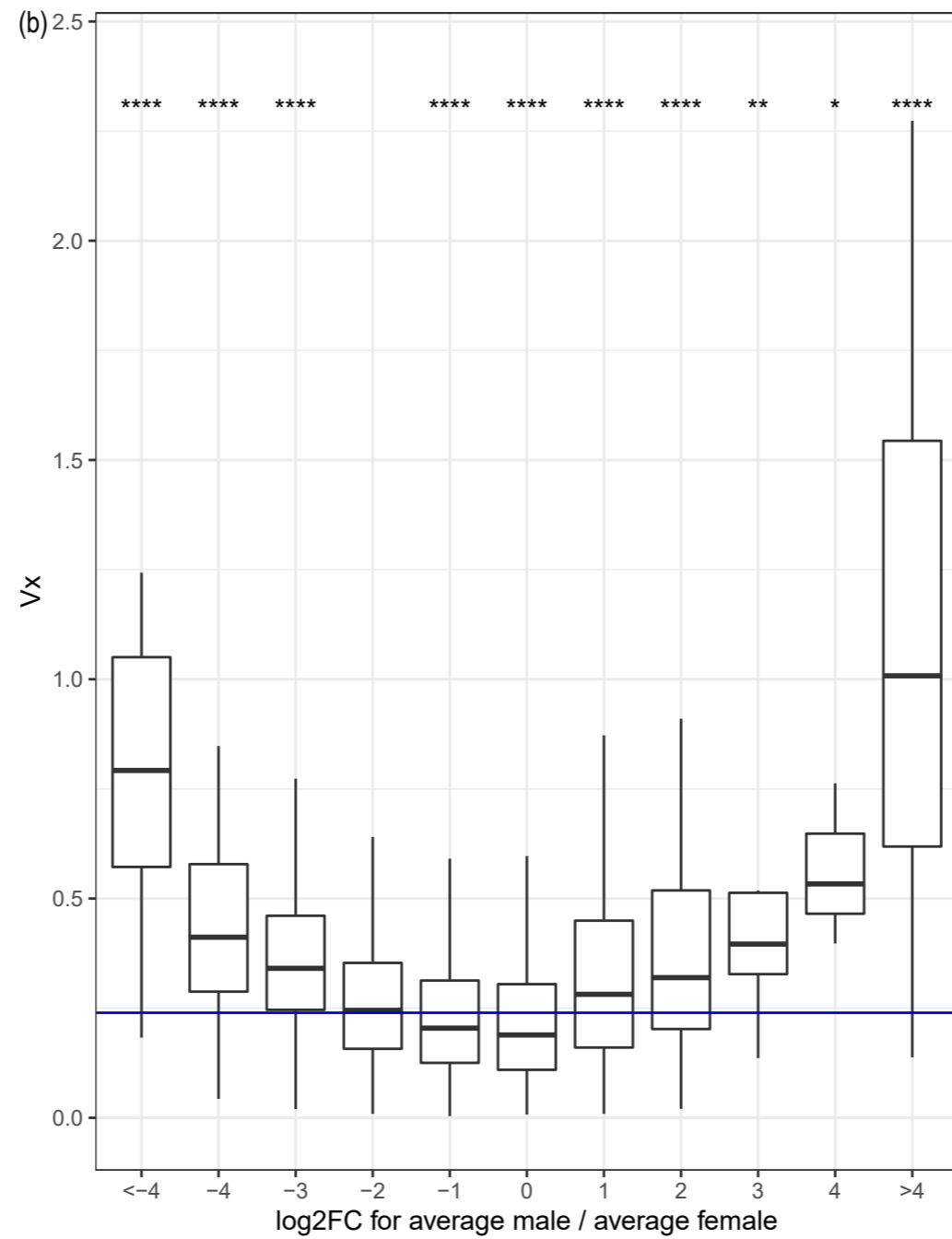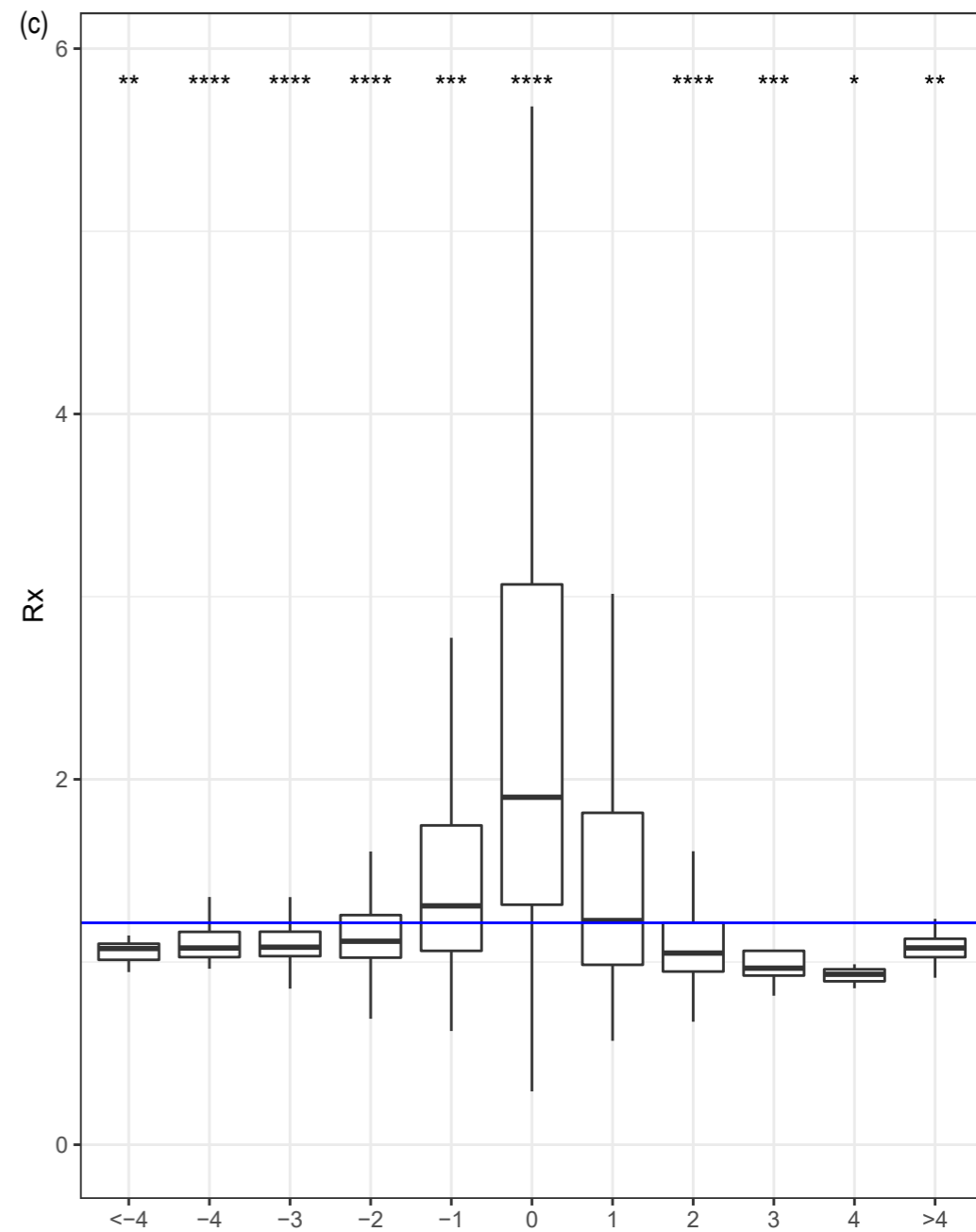

Supplement: Supplementary file 1 [file insects-11-00326-s001.zip › Fig. S3.pdf]

categories: Sex Sp Sex+Sp Int non-var

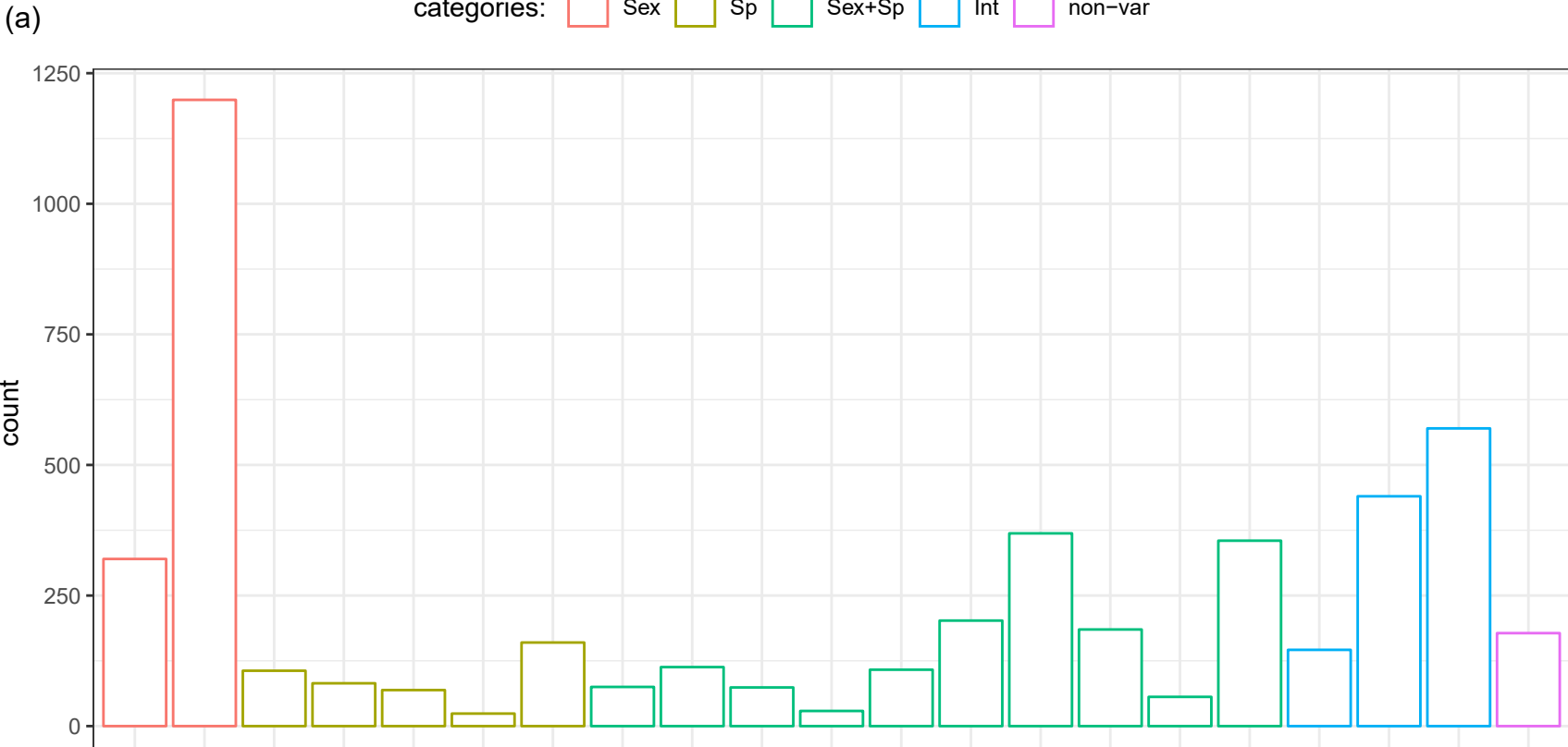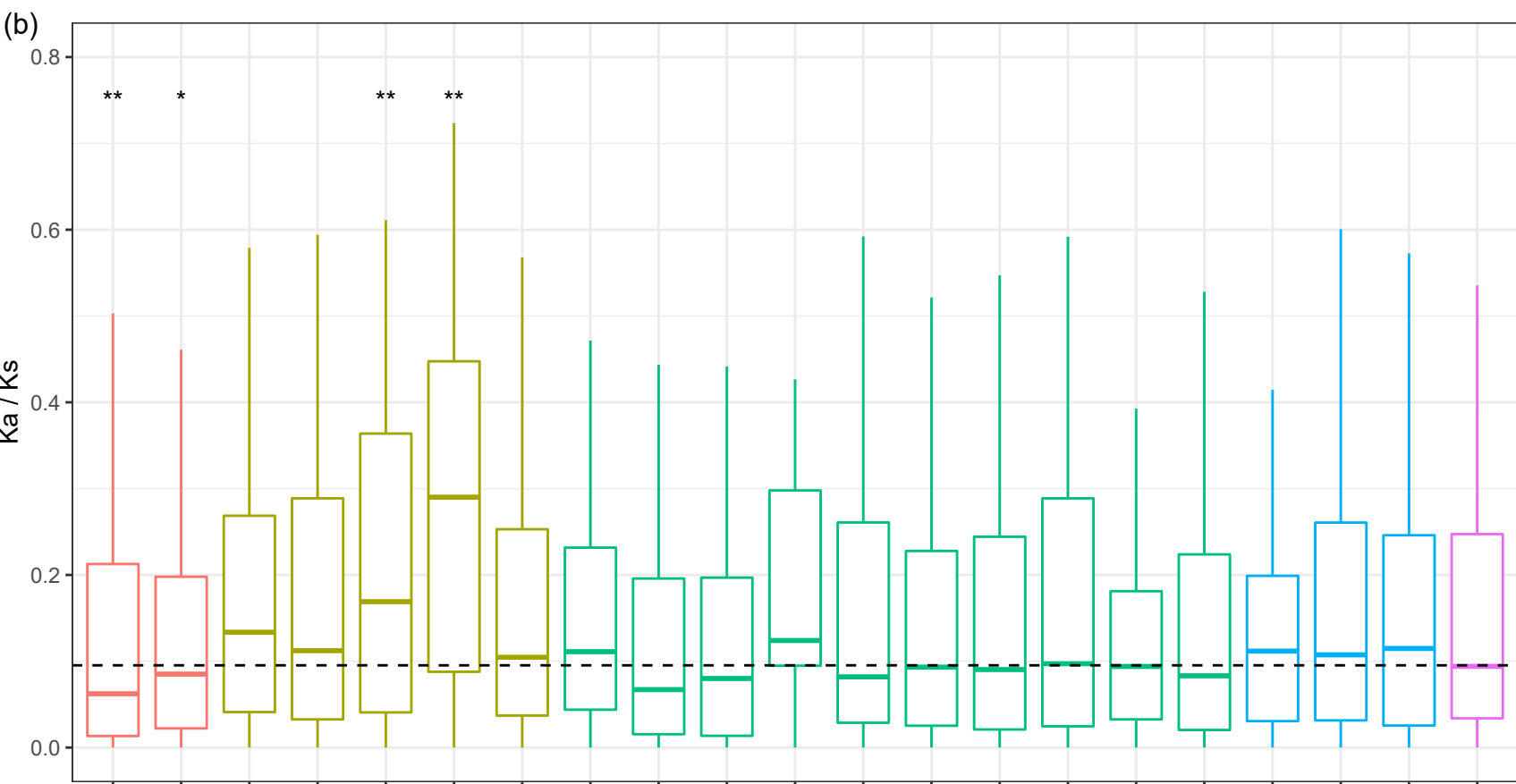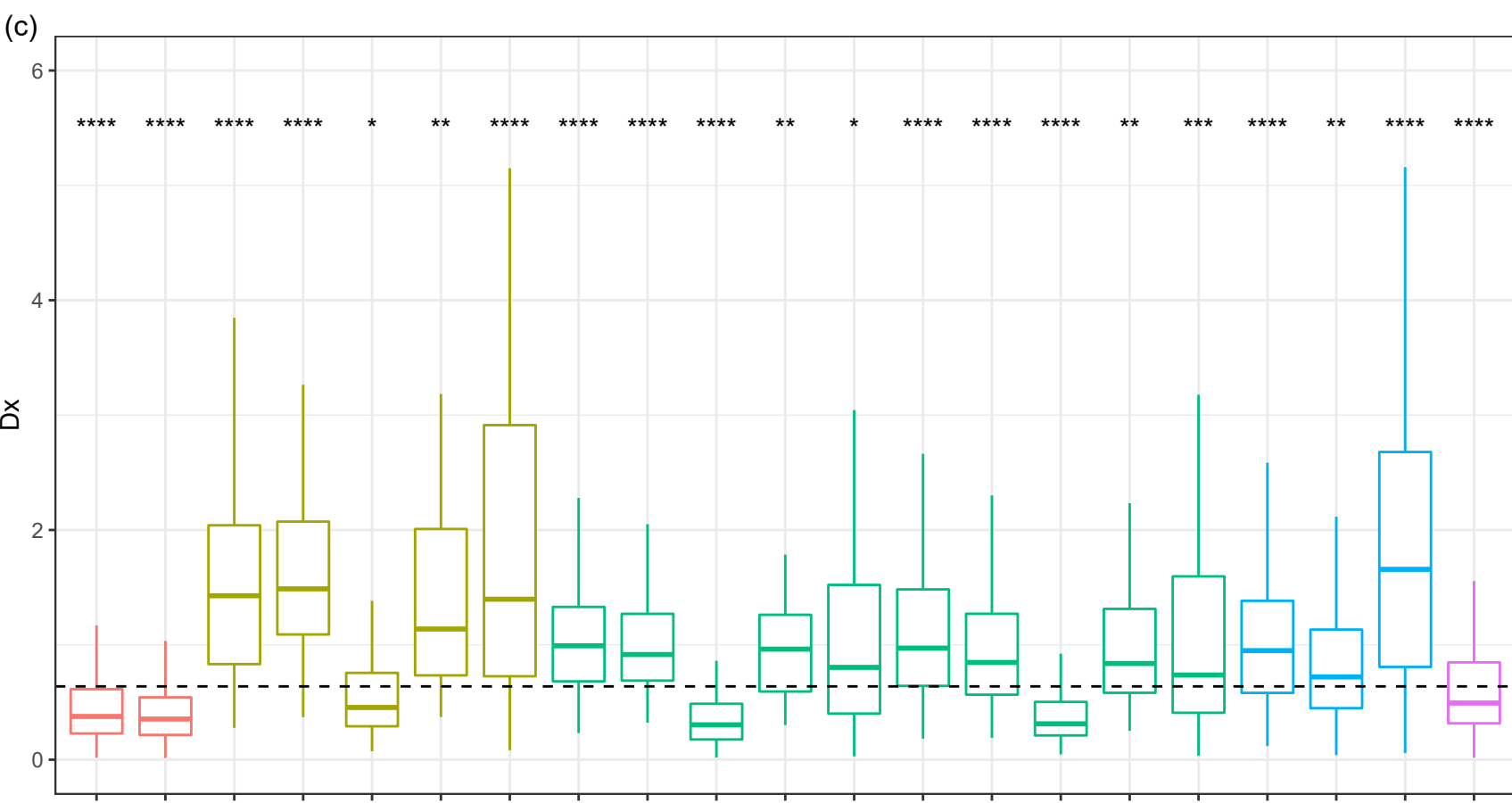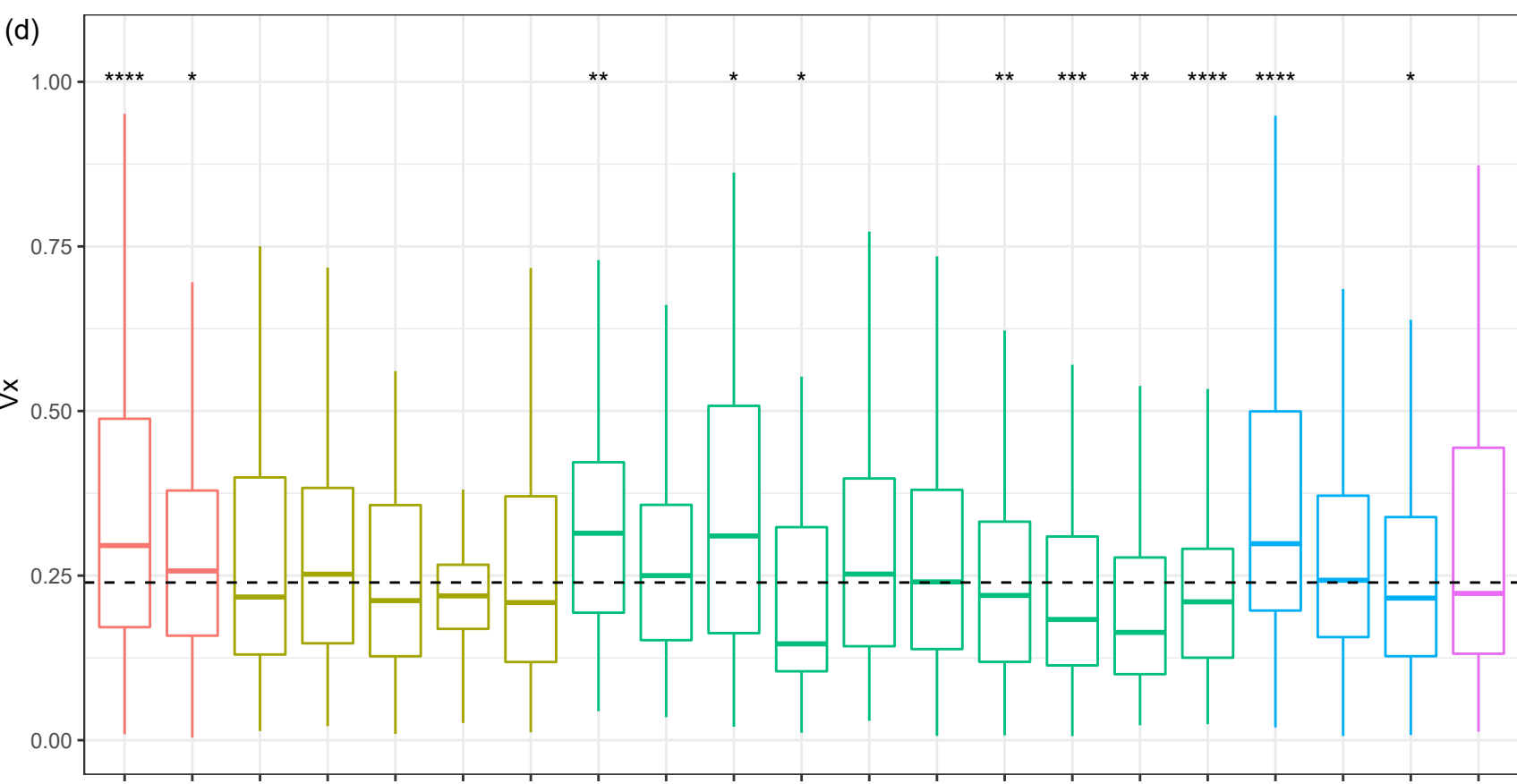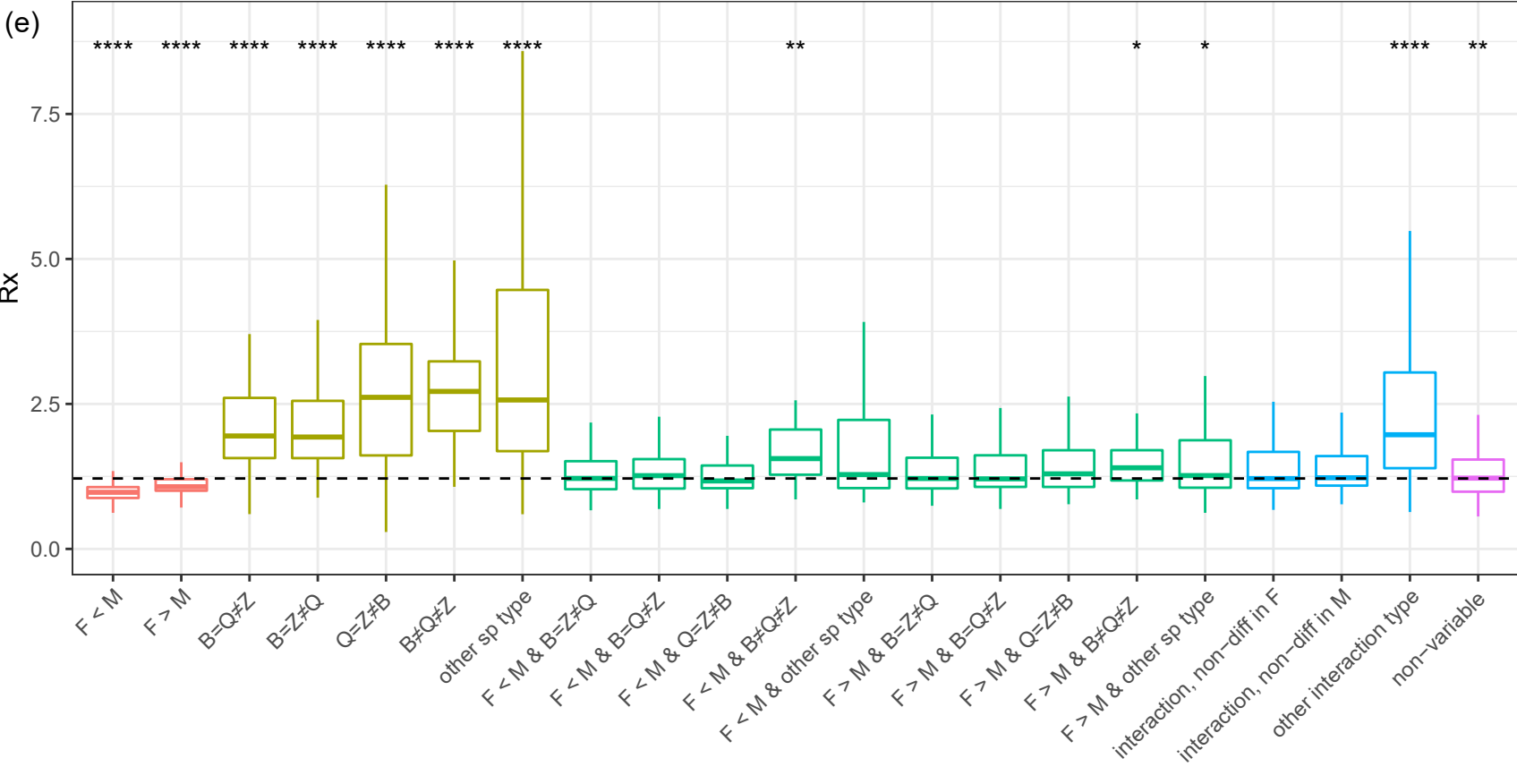

Supplement: Supplementary file 1 [file insects-11-00326-s001.zip › Fig. S4.pdf]
